# Epimedium-Derived Exosome-Loaded GelMA Hydrogel Enhances MC3T3-E1 Osteogenesis via PI3K/Akt Pathway

**DOI:** 10.3390/cells14151214

**Published:** 2025-08-07

**Authors:** Weijian Hu, Xin Xie, Jiabin Xu

**Affiliations:** 1 Medical College, Northwest University, Xi’an 710000, China; 202324229@stumail.nwu.edu.cn; 2 College of Life Sciences, Northwest University, Xi’an 710000, China; 3 School of Stomatology, Xuzhou Medical University, Xuzhou 221004, China

**Keywords:** gelatin methacryloyl, epimedium-derived exosomes, osteogenic differentiation, PI3K/Akt signaling pathway, anti-senescence, angiogenesis, sustained release

## Abstract

Healing large bone defects remains challenging. Gelatin scaffolds are biocompatible and biodegradable, but lack osteoinductive activity. Plant-derived exosomes carry miRNAs, growth factors, and proteins that modulate osteogenesis, but free exosomes suffer from poor stability, limited targeting, and low bioavailability in vivo. We developed a 3D GelMA hydrogel loaded with Epimedium-derived exosomes (“GelMA@Exo”) to improve exosome retention, stability, and sustained release. Its effects on MC3T3-E1 preosteoblasts—including proliferation, osteogenic differentiation, migration, and senescence—were evaluated via in vitro assays. Angiogenic potential was assessed using HUVECs. Underlying mechanisms were examined at transcriptomic and protein levels to elucidate GelMA@Exo’s therapeutic osteogenesis actions. GelMA@Exo exhibited sustained exosome release, enhancing exosome retention and cellular uptake. In vitro, GelMA@Exo markedly boosted MC3T3-E1 proliferation, migration, and mineralized nodule formation, while reducing senescence markers and promoting angiogenesis in HUVECs. Mechanistically, GelMA@Exo upregulated key osteogenic markers (RUNX2, TGF-β1, Osterix, COL1A1, ALPL) and activated the PI3K/Akt pathway. Transcriptomic data confirmed global upregulation of osteogenesis-related genes and bone-regeneration pathways. This study presents a GelMA hydrogel functionalized with plant-derived exosomes, which synergistically provides osteoinductive stimuli and structural support. The GelMA@Exo platform offers a versatile strategy for localized delivery of natural bioactive molecules and a promising approach for bone tissue engineering. Our findings provide strong experimental evidence for the translational potential of plant-derived exosomes in regenerative medicine.

## 1. Background

Osteoporosis is a metabolic bone disease characterized by low bone mass and microarchitectural deterioration, leading to weakened bone strength and high fracture risk [1]. It results from an imbalance between bone resorption by osteoclasts and bone formation by osteoblasts [2]. Approximately 80% of fragility fractures are osteoporosis-related [3]. Current therapies either stimulate osteoblast activity or inhibit osteoclasts [4], but long-term use of agents like estrogens or bisphosphonates can cause serious side effects (e.g., esophagitis, atypical fractures, osteonecrosis of the jaw) [5].

Plant-derived exosomes are 30–150 nm lipid-bilayer vesicles (a subclass of extracellular vesicles) [6,7] that carry microRNAs, proteins, and other bioactive factors that broadly promote tissue repair by stimulating cell migration, proliferation, and differentiation [8]. They also modulate inflammation, exhibit anti-tumor effects, enhance wound healing, and serve as natural nanocarriers for drug delivery [9]. Exosome-like nanoparticles from edible plants offer high biocompatibility, stability, longer half-life, and more efficient cellular uptake than mammalian or synthetic nanocarriers [10,11], making them promising bioactive molecule carriers in regenerative medicine [12]. However, free plant exosomes circulate only briefly and are rapidly cleared in vivo, limiting their sustained bioactivity [13]. An encapsulation strategy for stable, prolonged exosome delivery is needed.

Hydrogels are three-dimensional crosslinked polymer networks with excellent biocompatibility and biodegradability, widely used as scaffolds in tissue engineering [14]. Gelatin, a collagen-derived polymer, is inexpensive and biocompatible [15], and has a high capacity for loading bioactive molecules [16]. It contains Arg-Gly-Asp (RGD) motifs that promote cell adhesion [17]. To improve its stability and functionality, gelatin can be functionalized with methacryloyl groups to form gelatin methacryloyl (GelMA). Under UV light with a photoinitiator, GelMA crosslinks into a stable 3D hydrogel network [18,19]. Tuning the crosslink density allows precise control of GelMA’s mechanical strength, pore size, and degradation rate [20].

While hydrogels alone have limited bioactivity, embedding exosomes in a hydrogel can protect them and allow sustained, localized release, thereby amplifying their biological effects [21]. An ideal scaffold should mimic the natural extracellular matrix (ECM) to support cell growth and differentiation [22]. Recent strategies emphasize biomimetic, in situ-gelling hydrogels that integrate physical, chemical, and biological cues to create a regenerative microenvironment [23,24]. Incorporating exosomes into such scaffolds provides a stable delivery platform without the ethical or immunological issues of cell-based therapies, improving prospects for exosome-driven bone regeneration [25].

In summary, gelatin-based scaffolds are biocompatible and biodegradable, but lack osteoinductive activity, whereas plant-derived exosomes promote osteogenesis and angiogenesis, yet are unstable and inefficiently delivered in vivo. Combining exosomes with an ECM-mimicking hydrogel that offers injectability, tunable structure, and sustained release could create a more effective bone-regenerative microenvironment. Therefore, we constructed a photocrosslinkable GelMA hydrogel loaded with Epimedium-derived exosomes (GelMA@Exo) to leverage the combined advantages of hydrogels and exosomes. This study evaluates whether the GelMA@Exo system synergistically enhances MC3T3-E1 osteogenic differentiation in vitro, explores its potential in bone tissue engineering, and elucidates the molecular mechanisms underlying its osteoinductive effects.

## 2. Methods

### 2.1. Materials

Gelatin (EFL, Suzhou, China), LAP photoinitiator (EFL, Suzhou, China), Epimedium brevicornu leaves (wild-collected from Guiyang, Guizhou, China), Curing light source (EFL, Suzhou, China), SDS-PAGE kit (Umibio, Shanghai, China), PKH26 fluorescent dye (EFL, Suzhou, China), MC3T3-E1 preosteoblast cell line (Procell Life Science, Wuhan, China), DMEM medium (Procell Life Science, Wuhan, China), 0.25% Trypsin-EDTA solution (Yuanpei, Shanghai, China), β-glycerophosphate and ascorbic acid (Macklin, Shanghai, China), Dexamethasone (Abmole, Houston, TX, USA), CCK-8 assay kit (Enjing, Nanjing, China), ALP staining kit (Beyotime, Shanghai, China), Alizarin Red S staining kit (Solarbio, Beijing, China), Tube formation matrix gel (Abmole, Houston, TX, USA), Serum-free wound healing assay medium (Procell Life Science, Wuhan, China), SA-β-gal staining kit (Beyotime, Shanghai, China), RNA extraction and qPCR kits (Cowin Biosciences, Taizhou, China), LY294002 (Sigma-Aldrich, St. Louis, MO, USA), BCA protein quantification kit (Beyotime, Shanghai, China), BCA Protein Kit (Mele Biotech, Wuhan, China), PKH67 (Mele Biotech, Wuhan, China), Exosome isolation kit (Mele Biotech, Wuhan, China) and Exosome RNA extraction kit (Mele Biotech, Wuhan, China).

### 2.2. Epimedium Plant Material Processing and Exosome Isolation

Epimedium brevicornu leaves were rinsed with cold PBS and homogenized in PBS (1:1 *w*/*v*) containing protease inhibitors. The homogenate was sequentially centrifuged at 3000× *g* for 10 min and 10,000× *g* for 20 min at 4 °C to remove debris. The supernatant was filtered through a 0.22 μm membrane and ultracentrifuged at 100,000× *g* for 2 h (4 °C). The pellet was resuspended in PBS, ultracentrifuged again at 100,000× *g* for 1 h, and the final exosome pellet was collected and stored at −80 °C.

### 2.3. Preparation of Gelatin Hydrogels

Gelatin powder (EFL, China) was sterilized and dissolved in sterile PBS (pH 7.4) to prepare solutions at concentrations of 5%, 7.5%, 10%, and 12.5% (*w*/*v*). The solutions were magnetically stirred at 37 °C for 2 h to achieve homogeneous mixtures. To enable photocrosslinking, the photoinitiator lithium acetylacetonate (LAP, EFL, China) was added to the gelatin solution at a final concentration of 0.25% (*w*/*v*). The gelatin–LAP mixtures were then poured into molds and exposed to 365 nm UV light for 30 min under a biosafety cabinet to initiate crosslinking. Following crosslinking, the hydrogels were washed three times with sterile PBS (15 min per wash) to remove unreacted components and residual LAP. The hydrogels were then stored in fresh sterile PBS at 4 °C for subsequent use.

### 2.4. Preparation of GelMA@Exo (Exosome-Loaded GelMA Hydrogels)

Gelatin methacryloyl (GelMA) prepolymer solutions at concentrations of 5%, 7.5%, 10%, and 12.5% (*w*/*v*) in PBS were prepared by dissolving GelMA at 37 °C for 2 h. Epimedium-derived exosomes at 195 μg/mL were added to the GelMA solutions with different concentrations as described above, along with the photoinitiator lithium acylphosphinate (LAP) at 0.25% (*w*/*v*). The mixture was thoroughly mixed to ensure even distribution of exosomes and LAP. The exosome-loaded GelMA prepolymer was then cast into molds or 24-well plates and UV-crosslinked at 365 nm (approximately 10 cm distance) for 30 min to form GelMA@Exo hydrogels. After crosslinking, the hydrogels were washed three times with PBS (15 min per wash) to remove any unreacted monomers and unattached exosomes, and stored in PBS at 4 °C until further use.

This method is particularly effective for studying exosome release from GelMA@Exo hydrogels, as GelMA hydrogels offer tunable pore sizes that can be controlled by adjusting the GelMA concentration during preparation. This tunable porosity provides a controlled environment for exosome encapsulation and release, making it an ideal system for studying exosome dynamics and their therapeutic potential.

### 2.5. MC3T3-E1 Cell Culture and Osteogenic Induction

MC3T3-E1 preosteoblasts were maintained in complete DMEM and passaged at ~80% confluency. For osteogenic differentiation experiments, cells were seeded at 1 × 10^4^ cells/cm^2^ in 24-well plates. Upon reaching ~80% confluence, the medium was switched to osteogenic differentiation medium (DMEM supplemented with 10 mM β-glycerophosphate, 50 μg/mL ascorbic acid, and 100 nM dexamethasone). The medium was changed every 2–3 days. Cells were cultured under these conditions for 7–14 days, and samples were taken at specified time points for analysis of osteogenic markers.

### 2.6. Transwell Co-Culture with GelMA@Exo

To evaluate indirect effects of GelMA@Exo on cells, a Transwell co-culture system was used. MC3T3-E1 cells were seeded in the lower chamber of 24-well Transwells and allowed to attach for 24 h, and then the medium was replaced with osteogenic differentiation medium. A GelMA@Exo hydrogel (prepared as above) was placed in the Transwell insert (upper chamber), allowing exosomes to diffuse into the lower chamber without direct cell–hydrogel contact. This co-culture system enabled assessment of exosome-mediated effects on cell behavior (proliferation, differentiation, senescence, angiogenesis, etc.) while keeping the hydrogel physically separate from the cells.

### 2.7. Nanoparticle Tracking Analysis (NTA)

Purified exosome samples were passed through a 0.22 μm filter and diluted in PBS. Nanoparticle tracking analysis was performed at room temperature using a ZetaView^®^ instrument (Particle Metrix, Inning am Ammersee, Germany) equipped with a 405 nm laser. At least five fields were recorded (60 s videos each), and the particle size distribution (average diameter) and concentration were calculated using ZetaView software version 8.05.10. All measurements were done in triplicate.

### 2.8. Transmission Electron Microscopy (TEM)

For TEM, exosome suspensions (~1 × 10^10^ particles/mL) were applied (10 μL) onto carbon-coated copper grids and incubated for 5 min. Excess liquid was blotted off, and grids were stained with 2% phosphotungstic acid (pH 7.0) for 2 min. After air drying, the samples were examined with a HITACHI HT7700 transmission electron microscope at an accelerating voltage of 80–120 kV to observe exosome morphology.

### 2.9. Nucleic Acid Electrophoresis

Total RNA was extracted from exosome samples (e.g., using TRIzol) and quantified with a NanoDrop 2000 spectrophotometer (Thermo Fisher Scientific, Wilmington, DE, USA). For quality assessment, RNA samples were run on a 1.5% agarose gel in 1× TAE buffer at 100 V for ~25–30 min alongside a DNA ladder. RNA bands were visualized on a UV gel imaging system to confirm the presence of small RNA species.

### 2.10. SDS-PAGE Protein Analysis

Exosome proteins were extracted by lysing exosome pellets in RIPA buffer (Beyotime, Shanghai, China) containing protease inhibitors (30 min on ice). Lysates were centrifuged at 12,000× *g* for 20 min at 4 °C, and supernatants were collected. Protein concentration was determined by a BCA assay. For SDS-PAGE, 20 μg of exosomal protein per sample was loaded on 12% polyacrylamide gels and run at 120 V for ~1 h. Gels were then stained with Coomassie Brilliant Blue R-250 and destained in a standard acetic acid/methanol/water solution until protein bands were clear. Gel images were captured using a gel documentation system.

### 2.11. Fluorescent Labeling of Exosomes and Cellular Uptake

Epimedium-derived exosomes were labeled with the green fluorescent dye PKH67 (Mele Biotech, Wuhan, China) (following the manufacturer’s protocol; ~5 min incubation). The labeling was stopped with 1% BSA, and labeled exosomes were purified by ultracentrifugation (100,000× *g*, 70 min, 4 °C) and washing with PBS to remove free dye. The PKH67-labeled exosomes (195 μg/mL) were then mixed into a 5% (*w*/*v*) gelatin solution containing LAP and UV-crosslinked (365 nm, 30 min) to form fluorescently labeled exosome-loaded hydrogels. Separately, MC3T3-E1 cells were seeded in glass-bottom dishes (5 × 10^4^ cells/well). After cell attachment, the culture medium was replaced with fresh medium containing either the fluorescent GelMA@Exo hydrogel (in a Transwell insert) or an equivalent amount of released fluorescent exosomes. Cells were incubated with the fluorescent exosomes for 4 h or 12 h, then washed with PBS, fixed with 4% paraformaldehyde for 20 min, and counterstained with DAPI for nuclei. Exosome uptake by the cells was observed using a confocal laser scanning microscope (Leica TCS SP8, Leica Microsystems, Mannheim, Germany). Images (at excitation 488 nm for PKH67 and 405 nm for DAPI) from multiple fields were collected to qualitatively assess internalization of the green fluorescent exosomes (visible within the cytoplasm of cells, nuclei blue).

### 2.12. Scanning Electron Microscopy (SEM) of Hydrogels

GelMA hydrogels of various concentrations (5%, 7.5%, 10%, 12.5%) were prepared as described, then frozen and lyophilized for 48 h. The dried hydrogels were cut to expose their cross-sections, sputter-coated with gold, and imaged by SEM (Hitachi S-4800, Hitachi High-Technologies Corporation, Tokyo, Japan, 15 kV). SEM was used to examine the hydrogel surface and cross-sectional morphology, pore structure, and pore size distribution.

### 2.13. Swelling Behavior of GelMA Hydrogels

Lyophilized GelMA hydrogel samples (5%, 7.5%, 10%, 12.5% *w*/*v*) were weighed to determine the dry weight (W_d). Each sample was then immersed in PBS (pH 7.4, 37 °C). At predetermined time points (1, 2, 3, 4, 5, 6, 7, 8, 12 h), hydrogels were removed, quickly blotted to remove surface liquid, and weighed to obtain the swollen weight (W_s). The swelling ratio (%) was calculated as ((W_s − W_d)/W_d) × 100%. Each condition was tested in triplicate.

### 2.14. Degradation Behavior of GelMA Hydrogels

First, the lyophilized GelMA hydrogel was weighed and its dry weight (W_0_) was recorded. For hydrolytic degradation, the control sample was incubated in PBS (pH 7.4) at 37 °C with gentle shaking (80 rpm) for 14 days. For enzymatic degradation, the samples were incubated in PBS (pH 7.4) containing 1 mg/mL collagenase and incubated at 37 °C for the same time with gentle shaking (80 rpm). On days 1, 3, 5, 7, 10, and 14, the samples were removed, rinsed with PBS, lyophilized, and then weighed again to determine the remaining dry weight (W_t). The degradation rate (%) was calculated using the following formula: ((W_0_ − W_t)/W_0_) × 100%. All experiments were performed three times to ensure reproducibility and accurately characterize the hydrolytic and enzymatic degradation properties of GelMA hydrogels.

### 2.15. Distribution of Labeled Exosomes in GelMA Hydrogels

To visualize the spatial distribution of exosomes in the hydrogel matrix, Epimedium exosomes were fluorescently labeled with PKH67 (green) as above. Separately, a 5% (*w*/*v*) GelMA prepolymer solution was prepared containing 10 μg/mL of the red fluorescent dye PKH26. The green-labeled exosomes (195 μg/mL) were mixed into the red GelMA solution, which was then UV-crosslinked (365 nm, 30 min) in glass-bottom dishes to form a dual-labeled GelMA hydrogel (exosomes fluorescing green, hydrogel matrix red). Confocal laser scanning microscopy (Leica SP8) was used to image the hydrogels. Z-stack images (excitation 488 nm for PKH67, 561 nm for PKH26) were captured from three random fields per sample to reconstruct the 3D distribution of exosomes within the hydrogel. Co-localization of green and red signals indicated uniform embedding of exosomes throughout the GelMA network.

### 2.16. Exosome Release Kinetics from GelMA Hydrogels

Fluorescently labeled exosomes (PKH67) were incorporated into GelMA prepolymer solutions of 5%, 7.5%, 10%, and 12.5% and crosslinked to form GelMA@Exo hydrogels (as in Section 2.15). To assess release kinetics, equal-weight hydrogels were placed in individual wells with 2 mL PBS (pH 7.4) at 37 °C with shaking (80 rpm). At designated time points from day 1 to day 7, 0.5 mL of supernatant was collected and replaced with 0.5 mL of fresh PBS. The fluorescence intensity of released PKH67-labeled exosomes in each sample was measured (excitation 490 nm, emission 502 nm) using a microplate reader (BioTek Instruments, Winooski, VT, USA). Using a standard curve of fluorescence vs. exosome concentration, the cumulative exosome release (%) was calculated as (cumulative released exosome amount/total exosome amount loaded) × 100%.

### 2.17. Optimization of Exosome and GelMA Concentrations (CCK-8 Assays)

To determine the optimal exosome dosage for cell proliferation, MC3T3-E1 cells were seeded in 96-well plates (5 × 10^3^ cells/well). After 24 h, the medium was replaced with osteogenic differentiation medium containing 0, 65, 130, 195, or 260 μg/mL exosomes (n = 3 wells per group). Cell proliferation was assessed on days 1, 3, 5, and 7 using a CCK-8 assay: 10 μL of CCK-8 solution was added per well, incubated for ~1.5 h at 37 °C, and absorbance at 450 nm was measured. To evaluate the effect of gelatin concentration on cell growth, an indirect Transwell co-culture was used: MC3T3-E1 cells were cultured in the lower chambers of a 24-well plate while GelMA hydrogels of 5%, 7.5%, 10%, 12.5% were placed in the upper Transwell inserts. Cell proliferation was assessed over 7 days by CCK-8 in a similar manner. All experiments were performed in triplicate, and results are reported as mean ± SD.

### 2.18. Synergistic Effect of Gelatin Hydrogels and Exosomes on Cell Proliferation

To test the synergistic effect of combining GelMA and exosomes, four experimental groups were established: (1) ODM (osteogenic differentiation medium) control, (2) 5% GelMA hydrogel, (3) exosomes (195 μg/mL), and (4) 5% GelMA + 195 μg/mL exosomes (GelMA@Exo). MC3T3-E1 cells were seeded in 96-well plates (5 × 10^3^ cells per well in the lower chamber of Transwell inserts). After cell attachment, the respective treatment was added to the upper Transwell insert (a GelMA hydrogel, exosomes in medium, both, and none, depending on the group). At 0, 24, and 48 h, 10 μL of CCK-8 reagent was added to each well, incubated ~2 h, and absorbance at 450 nm was measured. Each condition was tested in triplicate (mean ± SD).

### 2.19. Alkaline Phosphatase (ALP) Staining

To assess early osteogenic differentiation, MC3T3-E1 cells were cultured under the four conditions (ODM, GelMA, Exo, GelMA@Exo). Cells were seeded in 24-well plates at 2 × 10^4^ cells/cm^2^ and treated as described. Medium was changed every 2–3 days. For experiments extending beyond one week, materials for different experimental groups were replenished every 7 days until the end of the experiment. At days 7 and 14, cells were washed with PBS and fixed with 4% paraformaldehyde for 20 min. ALP staining was performed using a BCIP/NBT alkaline phosphatase staining kit (Beyotime, Shanghai, China) according to the manufacturer’s instructions. After approximately 30 min incubation in the dark at room temperature, cells were gently washed and images of purple ALP staining were captured under an inverted microscope (Olympus IX73, Olympus Corporation, Tokyo, Japan). For semi-quantitative analysis of ALP activity, the area of ALP-positive staining in each well was measured using ImageJ software (version 1.53t, National Institutes of Health, Bethesda, MD, USA) and normalized to the total area of the well, yielding the percentage of area with ALP activity. Experiments were done in triplicate and results are expressed as mean ± SD.

### 2.20. Alizarin Red S Staining for Mineralization

MC3T3-E1 cells were seeded in 24-well plates at 2 × 10^4^ cells/cm^2^ andcultured in osteogenic medium under the four conditions (ODM, GelMA, Exo, GelMA@Exo) for up to 28 days. Medium was changed every 2–3 days. To ensure sustained exosome release over the long culture, materials for different experimental groups were replenished every 7 days until the end of the experiment. At days 21 and 28, cells were washed with PBS, fixed with 4% paraformaldehyde for 20 min, and stained with 2% Alizarin Red S (pH 4.2) for 15 min at room temperature. Excess dye was rinsed off with deionized water. Calcium mineral deposits appeared as red nodules, which were imaged under an inverted microscope. For quantification of mineralization, the bound dye was eluted by adding 500 μL of 10% cetylpyridinium chloride (CPC) to each well and incubating for 30 min. The optical density at 562 nm of the resulting solution was measured to quantify calcium content. The absorbance (OD_562) correlates with the amount of calcium deposited. All experiments were done in triplicate (mean ± SD reported).

### 2.21. Endothelial Tube Formation Assay

An in vitro tube formation assay was conducted to evaluate the pro-angiogenic effects of the treatments. HUVEC endothelial cells (5 × 10^4^ cells/well) were seeded on Matrigel-coated 48-well plates. Four treatment groups (each in triplicate) were applied: ODM control, 5% GelMA hydrogel, exosomes (195 μg/mL), and GelMA + exosomes. Matrigel (150 μL per well) was first added and allowed to solidify at 37 °C for 30 min. HUVECs were then plated on the Matrigel and overlaid with 300 μL of the respective conditioned medium or hydrogel extract. After 6 h incubation, capillary-like tube structures were visualized and photographed in three random fields per well using an inverted microscope. Tube formation was quantified using ImageJ: total tube length, number of junctions (nodes), and number of branches were measured for each image. The average values (mean ± SD) for each group were calculated to compare angiogenic activity.

### 2.22. Wound Healing (Cell Migration) Assay

MC3T3-E1 cells were grown to confluence (>90%) in 24-well plates. A linear scratch (“wound”) was made across each confluent monolayer using a sterile 200 μL pipette tip (Biosharp, Hefei, China). Cells were gently rinsed with PBS to remove debris and then incubated in serum-free osteogenic medium containing one of four treatments: ODM control, 5% GelMA hydrogel extract, exosomes (195 μg/mL), and GelMA + exosomes. Wound closure was monitored by photographing the wound area at 0, 24, and 48 h. The wound width was measured using ImageJ, and the percentage of wound closure was calculated as ((W_0 − W_t)/W_0) × 100%, where W_0 is the initial wound width and W_t is the width at time t. Triplicate wells were analyzed for each condition (results reported as mean ± SD).

### 2.23. SA-β-Gal Staining for Cellular Senescence

MC3T3-E1 cells were seeded in 24-well plates at 2 × 10^4^ cells/well and treated with ODM, 5% GelMA, 195 μg/mL exosomes, or GelMA + exosomes. Cellular senescence was evaluated on days 1, 4, and 7 using a Senescence β-Galactosidase Staining Kit (SA-β-Gal, Beyotime, Shanghai, China). On the specified days, cells were washed with PBS, fixed with the kit’s fixative solution for 15 min, then incubated with X-gal staining solution (pH 6.0) at 37 °C (in a CO_2_-free atmosphere) for ~16 h. After incubation, cells were rinsed with PBS and three random fields per well were imaged under a light microscope (Nikon Eclipse Ti2, Nikon Corporation, Tokyo, Japan). Senescent cells were identified by blue precipitate (SA-β-Gal positive staining). The proportion of senescent cells was calculated as (number of SA-β-Gal positive cells/total cell count) × 100%. Results (mean ± SD) were derived from three independent experiments.

### 2.24. Quantitative Real-Time PCR (qRT-PCR)

MC3T3-E1 cells were seeded in 6-well plates (2 × 10^5^ cells/well) and cultured for 14 days under the respective conditions (ODM, GelMA, Exo, GelMA@Exo). Total RNA was extracted using TRIzol™ reagent and checked for purity (A260/A280 ~1.8–2.0). One microgram of RNA per sample was reverse-transcribed into cDNA using a PrimeScript™ RT reagent kit (Takara Bio Inc., Kusatsu, Shiga, Japan) with gDNA eraser. Quantitative PCR was performed using SYBR Green PCR Master Mix (Yeasen Biotechnology, Shanghai, China) on a real-time PCR system. The osteogenic markers Runx2, Osterix (Sp7), Col1a1, Alpl, and Tgf-β1 were amplified, with GAPDH as the internal control. Relative gene expression levels were calculated using the ΔΔCt method. All reactions were run in triplicate. The data (mean ± SD) were analyzed for statistical significance as described below.

### 2.25. Western Blot Analysis

After 14 days of treatment under each condition, MC3T3-E1 cells were lysed in cold RIPA buffer containing protease inhibitors (30 min on ice). Lysates were centrifuged at 12,000× *g* for 15 min at 4 °C, and the supernatants were collected. Protein concentrations were measured via BCA assay. Equal amounts of protein (20–30 μg per sample) were separated on 10% SDS-PAGE gels and transferred onto PVDF membranes. Membranes were blocked with 5% non-fat milk in TBST for 1 h at room temperature, then incubated overnight at 4 °C with primary antibodies against Runx2, Osterix, COL1A1, TGF-β1, ALPL, and GAPDH (each at 1:1000 dilution). After washing in TBST, membranes were incubated with HRP-conjugated secondary antibodies (1:5000) for 1 h at room temperature. Protein bands were detected using ECL chemiluminescent reagents and imaged on a Bio-Rad system. Band intensities were quantified using ImageJ, normalized to the corresponding GAPDH band. Results from three independent experiments are expressed as mean ± SD.

### 2.26. Transcriptomic Sequencing and Pathway Analysis

To investigate the molecular mechanisms underlying GelMA@Exo’s osteoinductive effects, we performed transcriptomic (RNA-Seq) analysis and pathway validation. MC3T3-E1 cells were cultured for 7 days in osteogenic medium and without treatment (ODM control) and with GelMA@Exo (5% GelMA hydrogel containing 195 μg/mL exosomes). Total RNA was extracted (TRIzol) from each group, and high-quality RNA samples were used for library preparation and high-throughput sequencing (Illumina platform). After quality control (FastQC) and alignment to the mouse genome (e.g., with Hisat2), differential expression analysis was conducted (e.g., using DESeq2). Genes with |log_2(fold change)| > 1 and adjusted *p* < 0.05 were considered differentially expressed genes (DEGs). Gene Ontology (GO) enrichment and Kyoto Encyclopedia of Genes and Genomes (KEGG) pathway analysis were performed to identify biological processes and pathways significantly affected by GelMA@Exo. Particular attention was given to osteogenesis-related pathways; notably, PI3K/Akt signaling was found to be significantly upregulated in the GelMA@Exo group.

Based on the transcriptomic results, key signaling proteins were validated by Western blot. Specifically, the activation of the PI3K/Akt pathway was examined by measuring the levels of phosphorylated PI3K (p-PI3K) and phosphorylated AKT (p-AKT) in MC3T3-E1 cells treated with GelMA@Exo versus control. Additionally, the PI3K inhibitor LY294002 (Sigma-Aldrich, St. Louis, MO, USA) was used to confirm pathway involvement: GelMA@Exo-treated cells were also treated with LY294002 to see if blocking PI3K/Akt signaling would reduce the osteogenic effects. An increase in p-PI3K and p-AKT under GelMA@Exo treatment, and attenuation of this increase by LY294002, suggest that GelMA@Exo may mediate its osteogenic effect through PI3K/Akt activation.

Statistical analysis: all quantitative data are presented as mean ± standard deviation (SD). Statistical analysis was performed using one-way analysis of variance (ANOVA) followed by Tukey’s post hoc test for multiple comparisons. A *p*-value < 0.05 was considered statistically significant.

## 3. Construction and Biological Mechanism of the GelMA@Exosome System

**Figure 1:**
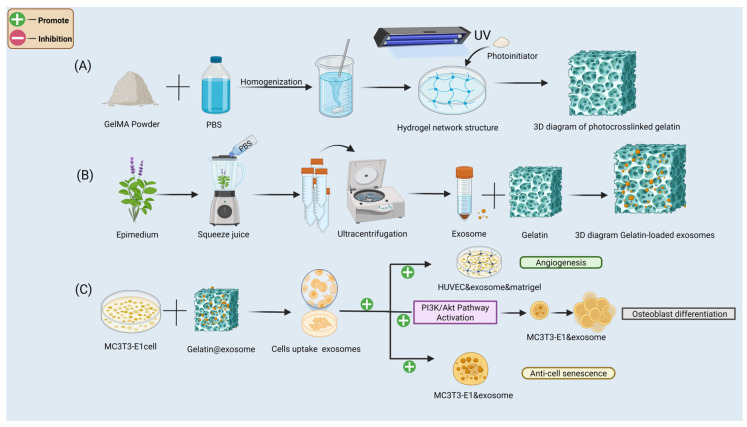
Schematic overview of the construction process and biofunctional mechanisms of the GelMA hydrogel-based delivery system encapsulating plant-derived exosomes. (**A**) Preparation of Photocrosslinked GelMA Hydrogel. (**B**) Isolation and Encapsulation of Plant-Derived Exsomes. (**C**) Biological Effects of the GelMA@Exosome System.

To provide a visual summary of the preparation process and biological mechanisms of the GelMA@Exosome hydrogel, a schematic diagram is presented in Figure 1. This figure outlines the stepwise construction of the delivery platform and its interactions with cells, including exosome uptake, angiogenesis, osteogenic differentiation, and anti-senescence effects.

(A) Preparation of photocrosslinked GelMA hydrogels: GelMA prepolymer (homogenization of methacryloyl gelatin in PBS with LAP photoinitiator) is UV-crosslinked to form a stable 3D porous network.
(B) Exosome isolation and loading: exosomes are extracted from Epimedium leaf tissues (by homogenization and ultracentrifugation) and then mixed into the GelMA prepolymer solution before UV-crosslinking to create the GelMA@Exo composite hydrogel.
(C) In vitro functional validation: the GelMA@Exo system promotes angiogenesis in HUVECs, activates the PI3K/Akt signaling pathway in MC3T3-E1 preosteoblasts, enhances osteogenic differentiation, and reduces cellular senescence.

A multifunctional 3D platform (GelMA@Exo) was thus designed to combine osteoinductive and regenerative properties. This system mimics the natural ECM, provides sustained exosome release, and modulates key cellular behaviors to create a pro-regenerative microenvironment for bone repair.

## 4. Results

### 4.1. Comprehensive Characterization of Epimedium-Derived Exosomes and GelMA Hydrogel Structure

**Figure 2:**
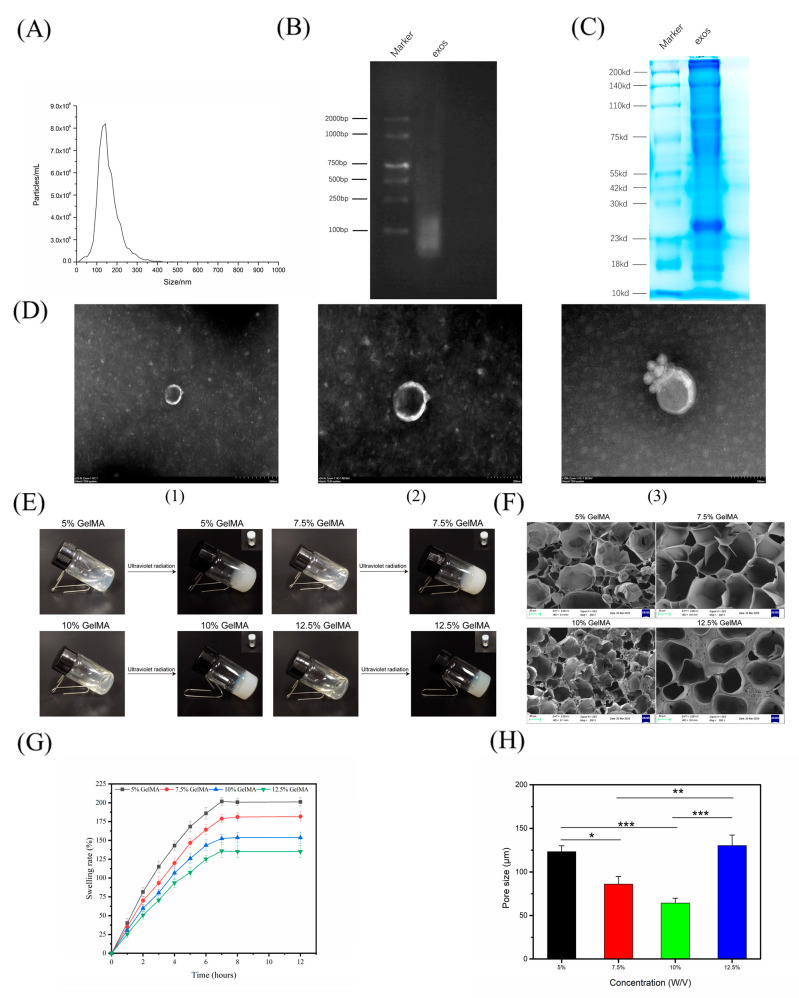
Comprehensive physicochemical characterization of Epimedium-derived exosomes and structural performance of GelMA hydrogels. (**A**) Size distribution of exosomes by nanoparticle tracking analysis (NTA). (**B**) Agarose gel electrophoresis of exosomal RNA. (**C**) SDS-PAGE protein profile of exosomes. (**D**) TEM image of exosomes (scale bar: 500 nm in D(1), 200 nm in D(2), and 100 nm in D(3)). (**E**) Macroscopic appearance of GelMA hydrogels at 5%, 7.5%, 10%, 12.5% concentrations (increasing GelMA concentration yields more opaque gels). (**F**) SEM micrographs of lyophilized GelMA hydrogels (scale bars: 50 μm) showing pore architecture at different concentrations. (**G**) Swelling ratio (%) of GelMA hydrogels over 12 h in PBS (mean ± SD, n = 3). (**H**) Average pore diameter of hydrogels measured from SEM images (mean ± SD, n = 3). Statistical analysis: one-way ANOVA with Tukey’s post hoc test. Significance: *** *p* < 0.001, ** *p* < 0.01, * *p* < 0.05.

Exosome characteristics: Nanoparticle Tracking Analysis (Figure 2A) showed that Epimedium-derived exosomes had a unimodal size distribution ranging from ~90 to 130 nm (average diameter ~103.6 ± 7.2 nm), consistent with typical exosome dimensions. Agarose gel electrophoresis of exosomal RNA (Figure 2B) displayed distinct bands around 100–250 bp and no visible 18S or 28S rRNA bands, indicating that the exosomal RNA consisted mainly of small RNAs and was free of significant contamination. SDS-PAGE analysis (Figure 2C) revealed prominent protein bands in the 23–100 kDa range, corresponding to expected exosomal proteins and confirming the high purity of the exosome preparation. Transmission electron microscopy (Figure 2D) showed the exosomes’ characteristic cup-shaped morphology with uniform size and intact membrane structure. Together, these results confirm that the isolated plant exosomes are of high quality (proper size, RNA content, and protein profile) for downstream biological applications.

Hydrogel structure: We next evaluated the structural properties of GelMA hydrogels at various concentrations. Macroscopically (Figure 2E), the hydrogels became increasingly opaque as GelMA concentration increased, suggesting higher crosslink density and smaller pore size at higher concentrations. SEM imaging (Figure 2F) confirmed that low-concentration GelMA hydrogels (5% and 7.5%) had large, well-distributed pores, whereas higher concentrations (10% and 12.5%) yielded a more compact microstructure with much smaller pores. In line with this, swelling tests (Figure 2G) showed that 5% GelMA absorbed significantly more water (e.g., ~206% swelling at 12 h) than 12.5% GelMA (~128% at 12 h), reflecting that higher gelatin concentration restricts swelling. Quantitative pore size analysis (Figure 2H) further demonstrated this trend: the average pore diameter in 10% GelMA (~62 μm) was significantly smaller than that of 5% or 7.5% gels, although the 12.5% gel showed a slight increase in mean pore size (likely due to network heterogeneity at very high polymer content).

In summary, the Epimedium exosomes exhibited appropriate nanoscale size, morphology, and molecular cargo, confirming their suitability for biological use. Concurrently, GelMA hydrogels showed concentration-dependent structural properties (pore size and swelling behavior), validating their role as tunable, stable carriers for encapsulating and delivering exosomes in a biomaterial system.

### 4.2. In Vitro Evaluation of Exosome Release and Cellular Uptake from GelMA Hydrogels

**Figure 3:**
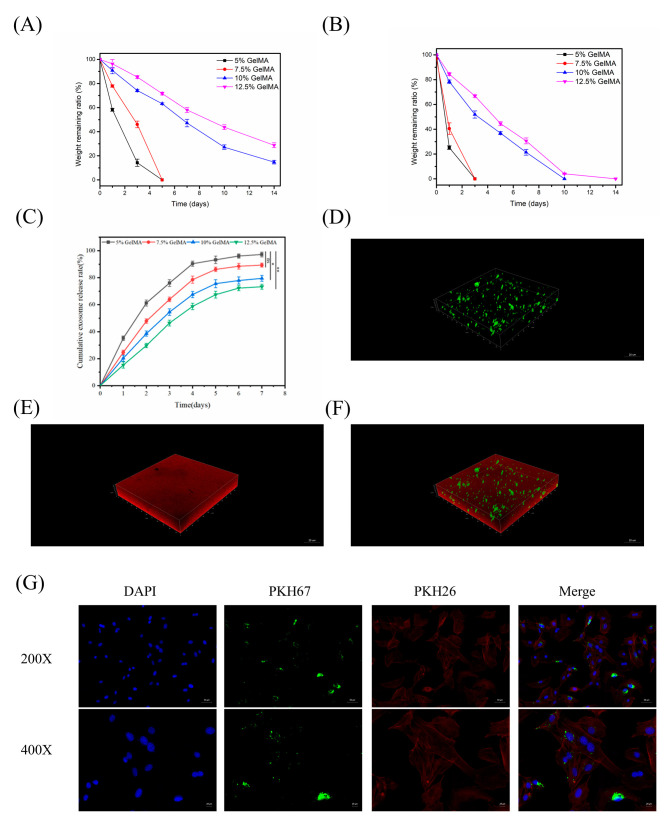
In vitro degradation, exosome release kinetics, and cellular uptake of GelMA hydrogels. (**A**) Degradation profiles of (5%, 7.5%, 10%, 12.5%) GelMA hydrogels in PBS over 14 days. (**B**) Enzymatic degradation of GelMA hydrogels (5–12.5%) in collagenase solution over 14 days. (**C**) Cumulative release of PKH67-labeled exosomes from GelMA@Exo hydrogels (5%, 7.5%, 10%, 12.5%) over 7 days (mean ± SD, n = 3). (**D**–**F**) 3D confocal images of PKH67-labeled exosomes (green) distributed in PKH26-labeled GelMA matrix (red) at different depths, showing uniform encapsulation of exosomes within the hydrogel. (**G**) Confocal image of MC3T3-E1 cells after co-culture with GelMA@Exo: green fluorescence inside cells indicates internalized exosomes (nuclei stained blue with DAPI). Statistical analysis: one-way ANOVA with Tukey’s post hoc test. Significance: ** *p* < 0.01, * *p* < 0.05; ns: not significant.

Degradation and release: GelMA hydrogels exhibited concentration-dependent degradability and exosome release profiles. In PBS (physiological conditions), 5% GelMA hydrogels lost the majority of their mass within ~5 days (Figure 3A), whereas 12.5% GelMA hydrogels degraded much more slowly, retaining structure beyond 7 days, indicating that higher GelMA concentrations yield more stable hydrogels. Similarly, under enzymatic conditions (collagenase, Figure 3B), higher GelMA concentration conferred greater resistance to degradation. Regarding exosome release, hydrogels with lower GelMA content released encapsulated exosomes more rapidly (Figure 3C). Notably, the 5% GelMA hydrogel released the largest cumulative amount of exosomes over 7 days, likely due to its larger pores and faster matrix degradation, whereas more densely crosslinked gels (10% and 12.5% GelMA) released exosomes at a slower rate. These results demonstrate that the GelMA concentration can be tuned to control the rate of exosome release, with lower GelMA percentages favoring faster release and higher percentages providing a more sustained release.

Cellular uptake: Confocal laser scanning microscopy confirmed that exosomes released from the hydrogels were effectively taken up by target cells. Three-dimensional confocal imaging of dual-labeled hydrogels (exosomes labeled green, GelMA matrix red) showed uniform distribution of exosomes throughout the GelMA scaffold with no obvious aggregation or premature leakage (Figure 3D–F). When MC3T3-E1 cells were co-cultured with GelMA@Exo hydrogels, substantial internalization of exosomes was observed: confocal images (Figure 3G) revealed green fluorescence within the red-stained cytoplasm of the cells (nuclei in blue), confirming that exosomes released from the GelMA matrix were efficiently taken up by the cells.

In summary, GelMA hydrogels degrade and release exosomes in a concentration-dependent manner: lower GelMA concentration (e.g., 5%) provides more rapid exosome release, whereas higher concentration (e.g., 10–12.5%) yields a slower, sustained release. Moreover, the exosomes delivered by the GelMA hydrogel remained bioactive and were readily internalized by MC3T3-E1 cells, underscoring the effectiveness of the GelMA@Exo platform for localized exosome delivery.

### 4.3. Synergistic Enhancement of Cell Proliferation, Angiogenesis, and Migration by GelMA@Exo

**Figure 4:**
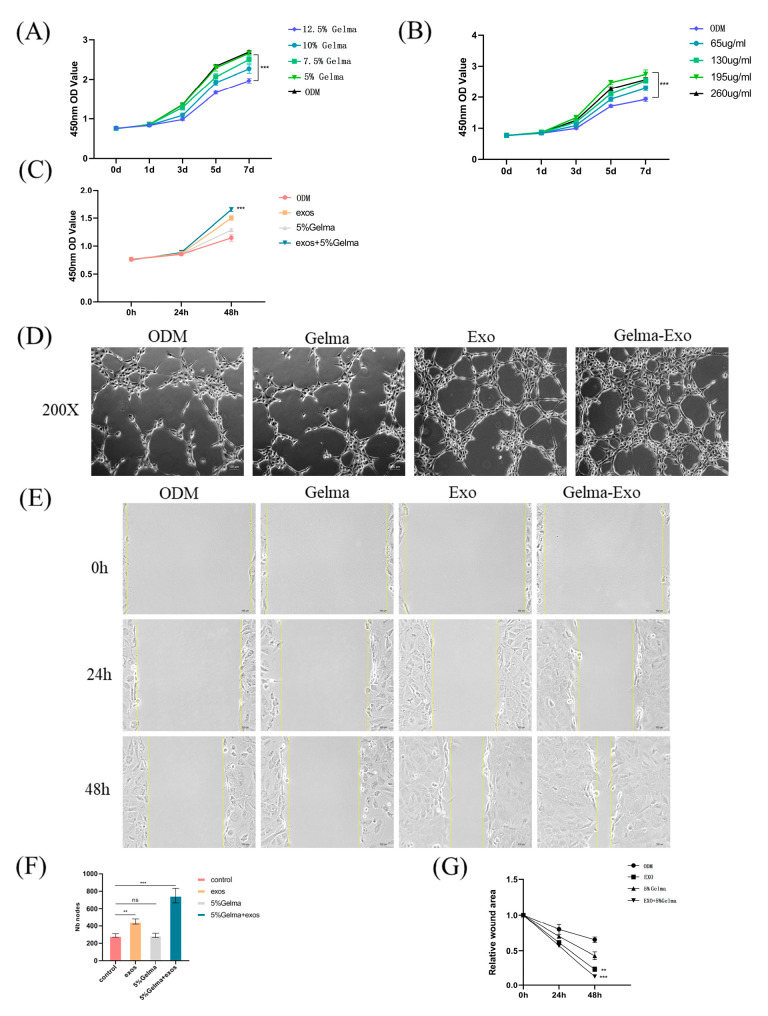
Synergistic effects of GelMA hydrogel and Epimedium-derived exosomes on MC3T3-E1 proliferation, migration, and HUVEC angiogenesis. (**A**–**C**) MC3T3-E1 cell proliferation under varying conditions (GelMA concentration, exosome dose, and their combination) measured by CCK-8 assay over 7 days (mean ± SD, n = 3). (**D**) Representative images of HUVEC tube formation in each group after 6 h of incubation (scale bar: 100 μm). (**E**) Representative images of scratch wound healing in MC3T3-E1 cells at 0, 24 h, and 48 h under each treatment (scale bar: 100 μm). (**F**) Quantification of tube formation (number of nodes) for HUVEC networks (mean ± SD, n = 3; GelMA@Exo vs. others). (**G**) Quantification of wound closure percentage at 24 h and 48 h in the migration assay (mean ± SD, n = 3). Statistical analysis: one-way ANOVA with Tukey’s post hoc test. Significance: *** *p* < 0.001, ** *p* < 0.01; ns: not significant.

Cell proliferation: In CCK-8 assays, GelMA hydrogel alone had minimal impact on MC3T3-E1 cell proliferation over 7 days (in fact, higher GelMA concentrations slightly suppressed proliferation), whereas Epimedium exosomes alone increased cell proliferation in a dose-dependent manner, with an optimal concentration around 195 μg/mL. Based on these results, we chose 5% GelMA combined with 195 μg/mL exosomes as the GelMA@Exo condition for synergy tests. This composite condition showed a clear synergistic effect: MC3T3-E1 cells exposed to GelMA@Exo displayed significantly higher proliferation at 24 h and 48 h compared to cells treated with GelMA alone, exosomes alone, or ODM (Figure 4A–C). Thus, GelMA@Exo outperformed the individual components in promoting osteoblast proliferation.

Angiogenesis: In vitro tube formation assays using HUVECs demonstrated that exosome treatment alone promoted the formation of capillary-like networks, whereas 5% GelMA alone had no pro-angiogenic effect (comparable to ODM). Notably, the GelMA@Exo combination led to the most robust angiogenic response: HUVECs formed dense, highly interconnected tube networks in the GelMA@Exo group (Figure 4D). Quantification of tube network nodes (junctions) and total length (Figure 4F) confirmed that GelMA@Exo significantly increased angiogenic metrics (*** *p* < 0.001 vs. control (ODM) groups for node count), while there was no significant difference between GelMA-alone and ODM groups. These results indicate that sustained release of exosomes from the hydrogel markedly enhances angiogenesis, an effect not achievable by the hydrogel alone.

Cell migration: A scratch wound healing assay was used to assess cell migration. MC3T3-E1 monolayers treated with GelMA@Exo exhibited much faster wound closure compared to other groups. By 24 h and 48 h, the GelMA@Exo-treated cells had filled a significantly greater portion of the wound gap (Figure 4E). Quantitative analysis of wound closure rates (Figure 4G) showed that the GelMA@Exo group achieved the highest percentage of wound closure at both 24 and 48 h, whereas exosomes alone had a moderate effect and GelMA alone was similar to ODM. This demonstrates that the combination of hydrogel and exosomes synergistically accelerates osteoblast migration.

In summary, the GelMA@Exo composite hydrogel markedly enhanced MC3T3-E1 cell proliferation, promoted angiogenic tube formation by endothelial cells, and accelerated osteoblast migration. These synergistic effects highlight the potential of GelMA@Exo as a multifunctional scaffold to support vascularized bone regeneration, outperforming the efficacy of hydrogel or exosomes alone.

### 4.4. GelMA@Exo Enhances Late-Stage Osteogenic Mineralization

**Figure 5:**
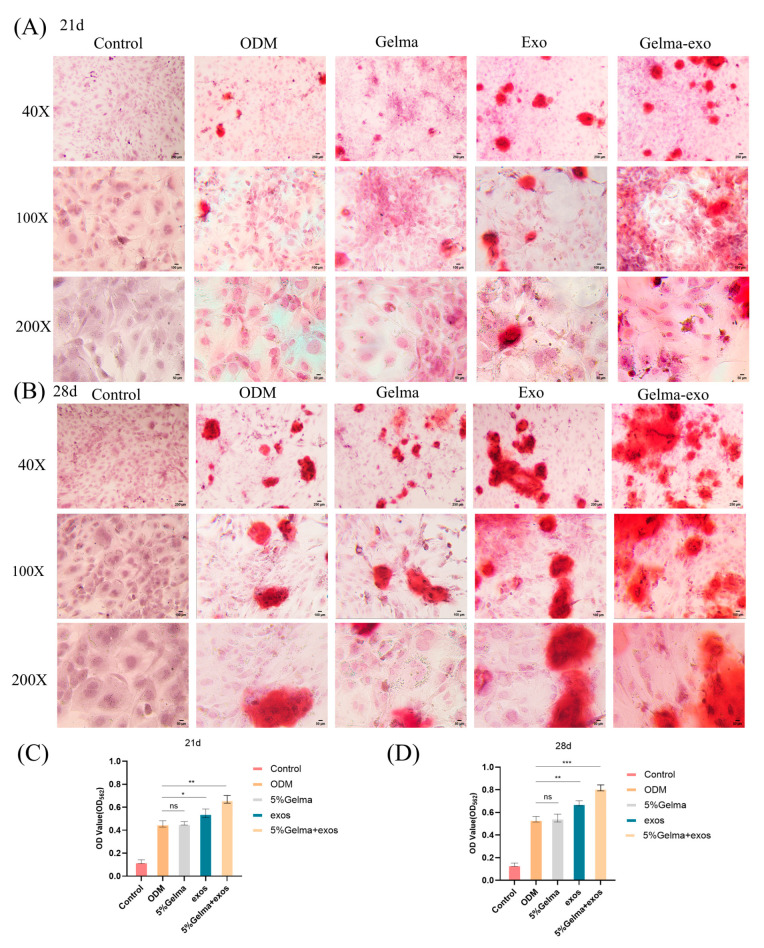
GelMA@Exo promotes mineralized matrix formation in MC3T3-E1 cells. (**A**,**B**) Alizarin Red S staining of mineralized nodules in different groups on day 21 (**A**) and day 28 (**B**). Images are shown at 40× (scale bar: 250 μm), 100× (scale bar: 100 μm), and 200× (scale bar: 50 μm) magnification. GelMA@Exo exhibits the most extensive mineralization (deep red nodules) at both time points. (**C**,**D**) Quantification of calcium deposition (OD_562 values from CPC extraction) for day 21 (**C**) and day 28 (**D**). Data are mean ± SD (n = 3);. Statistical analysis: one-way ANOVA with Tukey’s post hoc test. Significance: *** *p* < 0.001, ** *p* < 0.01, * *p* < 0.05; ns: not significant.

To determine the impact on matrix mineralization (a late-stage indicator of osteogenesis), Alizarin Red S (ARS) staining was conducted after 21 and 28 days of induction. Day 21: As expected, the uninduced control showed no mineralized nodules, and the ODM-only group showed only sparse mineral deposition. GelMA hydrogel alone was similar to ODM, indicating it did not enhance mineralization by itself. In contrast, exosome treatment alone led to extensive red staining with numerous mineralized nodules, demonstrating that Epimedium exosomes significantly promote mineralization. Importantly, the GelMA@Exo composite produced the most intense ARS staining and the highest density of calcium nodules at day 21 (Figure 5A), indicating that the sustained delivery of exosomes via the hydrogel greatly enhanced osteogenic mineralization compared to all other groups. Day 28: All induced groups exhibited more mineral deposition at 28 days than at 21 days (Figure 5B). GelMA@Exo continued to yield the most robust mineralization, with large, coalescing mineral nodules evident throughout the wells. The exosome-only group also showed substantially higher mineralization than the GelMA-alone or ODM groups. GelMA alone remained comparable to ODM (showing no significant improvement in mineralization), underscoring that the hydrogel requires the exosome payload to impart osteoinductive effects.

Quantitative analysis of calcium accumulation via cetylpyridinium chloride extraction and absorbance measurement corroborated the qualitative staining results (Figure 5C,D). GelMA@Exo showed the highest calcium content at both day 21 and day 28, confirming a strong synergistic effect on mineralized matrix formation. Exosomes alone also significantly increased calcium deposition compared to GelMA alone or ODM whereas GelMA alone did not differ from the ODM baseline (as expected, the non-induced control had minimal calcium at both time points). These results demonstrate that continuous delivery of plant exosomes from the GelMA scaffold dramatically enhances late-stage osteogenic differentiation, as evidenced by calcium-rich mineralized matrix production.

### 4.5. GelMA@Exo Enhances Early Osteogenic Differentiation (ALP Activity)

**Figure 6:**
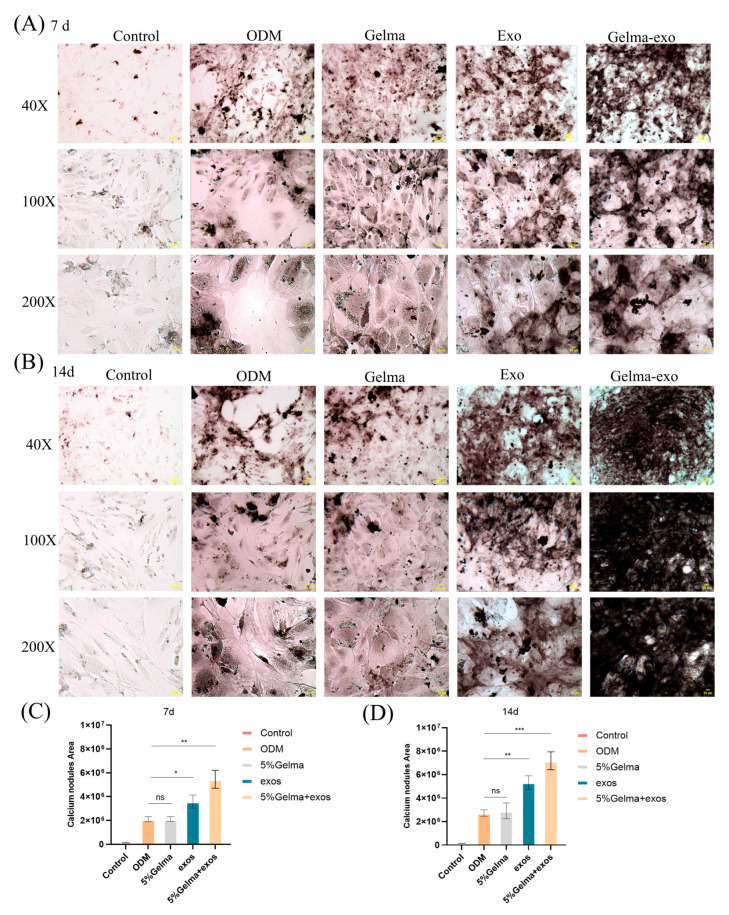
Evaluation of early osteogenic differentiation via ALP staining. (**A**,**B**) Representative ALP staining images of MC3T3-E1 cells on day 7 (**A**) and day 14 (**B**) under different conditions 40× (scale bar: 250 μm), 100× (scale bar: 100 μm), 200× (scale bar: 50 μm) magnification. Black staining indicates ALP activity; GelMA@Exo-treated cells show the most ALP-positive area at both time points. (**C**,**D**) Quantification of ALP activity (as percentage of area stained or absorbance) on day 7 (**C**) and day 14 (**D**). Data are mean ± SD (n = 3); Statistical analysis: one-way ANOVA with Tukey’s post hoc test. Significance: *** *p* < 0.001, ** *p* < 0.01, * *p* < 0.05; ns: not significant.

We next evaluated early-stage osteogenic differentiation using alkaline phosphatase (ALP) staining on days 7 and 14. Day 7: In the absence of osteogenic induction (control), MC3T3-E1 cells showed no ALP staining. Cells in ODM (induction medium alone) displayed only faint ALP staining, indicating baseline differentiation. GelMA hydrogel alone did not increase ALP staining beyond the ODM baseline, confirming that GelMA by itself is not osteoinductive. In contrast, exosome treatment alone resulted in substantially more ALP-positive areas (intense purple staining), demonstrating that Epimedium exosomes significantly upregulate early osteogenic activity. The GelMA@Exo composite yielded the strongest ALP staining of all groups (Figure 6A), indicating a synergistic enhancement of early differentiation (more ALP production than exosomes alone). Day 14: ALP activity increased in all groups by day 14 (Figure 6B). The GelMA@Exo group still showed the most extensive ALP staining, reflecting sustained high ALP activity. Exosomes alone also continued to produce more ALP staining than GelMA or ODM. GelMA alone remained similar to ODM (no marked improvement). This consistent pattern confirms that the presence of exosomes is crucial for stimulating early osteogenic differentiation, and the GelMA scaffold further amplifies this effect.

Quantitative ALP assay data (measuring the stained area or extracted dye optical density) aligned with the visual observations (Figure 6C,D). At day 7, GelMA@Exo had the highest ALP level (** *p* < 0.01 vs. ODM groups), while exosomes alone also significantly increased ALP activity compared to ODM or GelMA-only (* *p* < 0.05). GelMA alone did not differ from the ODM. By day 14, GelMA@Exo still showed the greatest ALP activity (*** *p* < 0.001), and exosomes alone remained significantly higher than GelMA or ODM (** *p* < 0.01). GelMA without exosomes had negligible effect throughout. Thus, only treatments containing exosomes—particularly the GelMA@Exo composite—robustly enhanced ALP activity, demonstrating an accelerated early differentiation that gelMA could not achieve alone.

### 4.6. GelMA@Exo Attenuates Cellular Senescence and Upregulates Osteogenic Gene Expression

**Figure 7:**
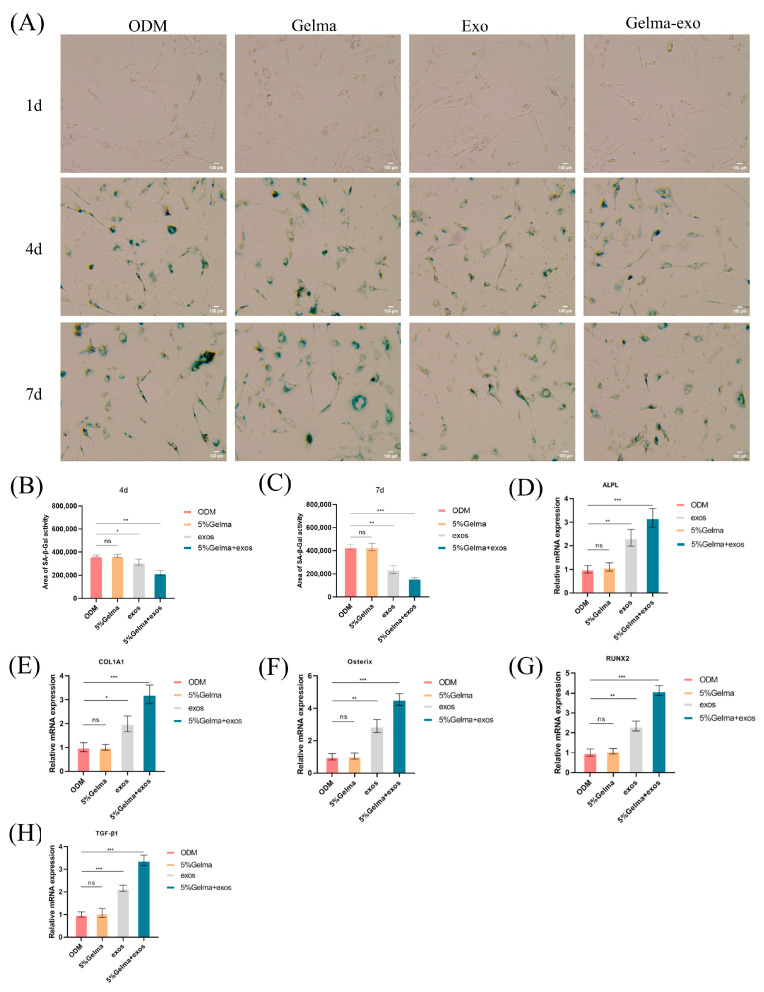
GelMA@Exo’s effects on cellular senescence and osteogenic gene expression. (**A**) SA-β-Gal staining images of MC3T3-E1 cells on days 1, 4, 7 for each group (green-blue staining indicates senescent cells; scale bar: 100 μm). (**B**,**C**) Quantification of SA-β-Gal-positive cells on day 4 (**B**) and day 7 (**C**) (mean ± SD, n = 3). GelMA@Exo shows the lowest senescent cell percentage (**D**–**H**) Relative mRNA expression of osteogenic genes (ALPL, Osterix, COL1A1, TGF-β1, RUNX2) after 7 days of treatment, measured by qRT-PCR (mean ± SD, n = 3). GelMA@Exo significantly upregulates all these genes compared toODM. Statistical analysis: one-way ANOVA with Tukey’s post hoc test. Significance: *** *p* < 0.001, ** *p* < 0.01, * *p* < 0.05; ns: not significant.

We investigated whether GelMA@Exo could reduce cellular senescence in osteoblasts and how it affects osteogenic gene expression. Cellular senescence: Senescence-associated β-galactosidase (SA-β-Gal) staining was used as a marker for aging cells. At day 4 and day 7, the ODM and GelMA-alone groups showed a substantial number of SA-β-Gal-positive (blue) cells, indicating the presence of senescent cells under normal induction conditions. In contrast, the exosome-treated groups (exosomes alone and GelMA@Exo) had markedly fewer blue cells (Figure 7A). Quantification (Figure 7B,C) revealed that GelMA@Exo-treated cultures had the lowest percentage of senescent cells at both day 4 and 7. The exosome-only group also showed a significant reduction in senescent cells compared to ODM or GelMA, though not as pronounced as GelMA@Exo. These results suggest that Epimedium exosomes can delay or reduce osteoblast senescence, and the GelMA hydrogel delivery further enhances this anti-senescence effect, likely by maintaining a higher local concentration of exosomes over time.

Osteogenic gene expression: We measured the mRNA levels of key osteogenic markers (Runx2, Osterix, COL1A1, ALPL, and TGF-β1) by qRT-PCR after 7 days of treatment (Figure 7D–H). GelMA@Exo treatment led to significant upregulation of all examined osteogenic genes compared to ODM, GelMA alone, or exosomes alone. In particular, GelMA@Exo strongly increased the early transcription factors Runx2 and Osterix, which drive osteoblast differentiation, as well as Col1a1 and Alpl, which are markers of matrix production and mineralization. Notably, Tgf-β1 expression was dramatically elevated in the GelMA@Exo group (much higher than in exosome-alone treatment), implicating activation of TGF-β signaling pathways. Exosomes alone also upregulated these genes to a lesser degree (significantly vs. GelMA or control(ODM) in some cases), whereas GelMA alone had little to no effect on gene expression.

In summary, the GelMA@Exo composite not only rejuvenated the osteoblast culture by reducing cellular senescence, but also induced a broad osteogenic gene expression program. The plant-derived exosomes remained highly bioactive when delivered via GelMA, and the combination synergistically created a more pro-osteogenic, anti-senescent microenvironment than either component alone. These molecular findings align with the functional assays, reinforcing the conclusion that GelMA@Exo provides multifaceted support for osteogenic differentiation.

### 4.7. GelMA@Exo Synergistically Upregulates Osteogenic Protein Expression

**Figure 8:**
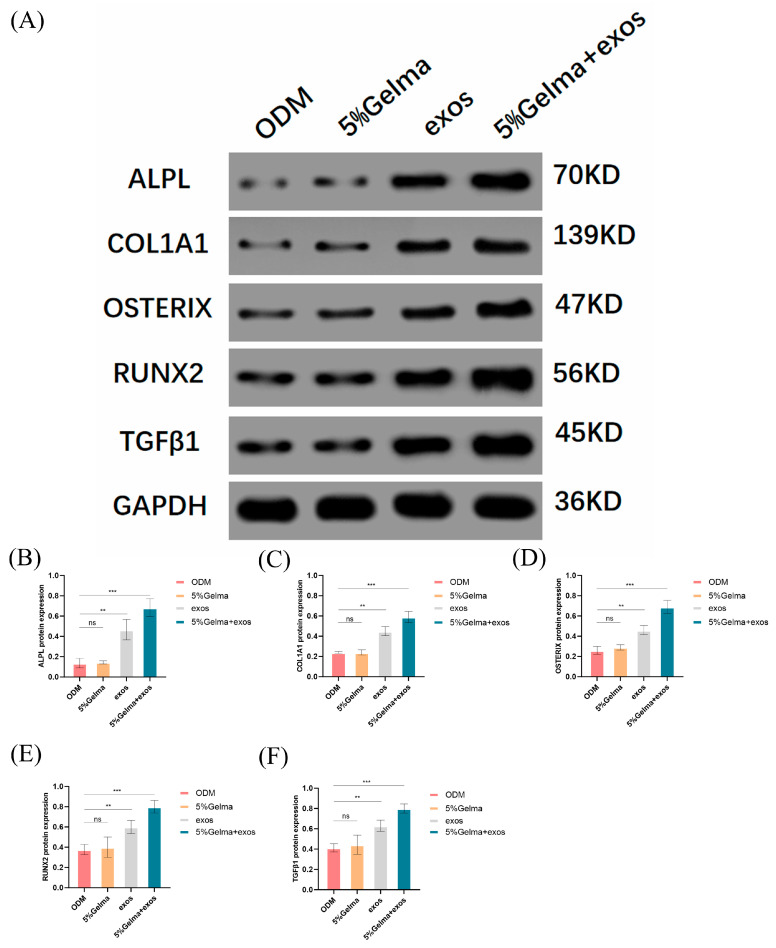
Osteogenic protein expression and quantification under different treatments. (**A**) Western blot detection of osteogenic proteins (ALPL, COL1A1, Osterix, RUNX2, TGF-β1) in MC3T3-E1 cells after 14 days of treatment with control (ODM), GelMA (5%), Exosomes, or GelMA@Exo. GAPDH serves as the loading control. (**B**–**F**) Densitometric analysis of Western blots for ALPL (**B**), COL1A1 (**C**), Osterix (**D**), RUNX2 (**E**), and TGF-β1 (**F**) (mean ± SD, n = 3). Statistical analysis: one-way ANOVA with Tukey’s post hoc test. Significance: *** *p* < 0.001, ** *p* < 0.01; ns: not significant. GelMA@Exo yields the highest expression for all markers, demonstrating a synergistic enhancement over exosomes or GelMA alone.

To validate whether the observed gene expression changes translated into corresponding alterations at the protein level, we performed Western blot analysis to examine key osteogenic markers, including ALPL, COL1A1, Osterix, RUNX2, and TGF-β1 (Figure 8A–F). Both the ODM control and 5% GelMA-alone groups exhibited only baseline protein expression, without significant differences observed between these two conditions. Treatment with exosomes alone significantly enhanced the expression of all analyzed osteogenic proteins compared to the ODM control group, clearly indicating the osteoinductive capacity of exosomes. Notably, the combined GelMA@Exo treatment yielded the highest protein expression levels across all markers. Specifically, ALPL and COL1A1 levels were markedly elevated in the GelMA@Exo group relative to the exosome-alone group. Similarly, critical transcription factors for osteogenesis, Osterix and RUNX2, showed significantly higher expression in the GelMA@Exo group compared to either treatment alone. Furthermore, the TGF-β1 protein, essential for osteogenic differentiation signaling, was notably upregulated with the combined treatment.

In summary, Western blot data mirrored the osteogenic protein expression findings: Exosomes alone substantially increased osteogenic protein levels, and the GelMA@Exo composite further maximized these levels beyond what exosomes alone achieved. Neither the scaffold nor exosomes by themselves matched the efficacy of the combined system. Thus, incorporating Epimedium exosomes into a GelMA hydrogel provides a highly effective strategy to upregulate osteogenic markers at protein level, corroborating the enhanced differentiation observed functionally.

### 4.8. Transcriptomic Profiling of GelMA@Exo-Treated Cells

**Figure 9:**
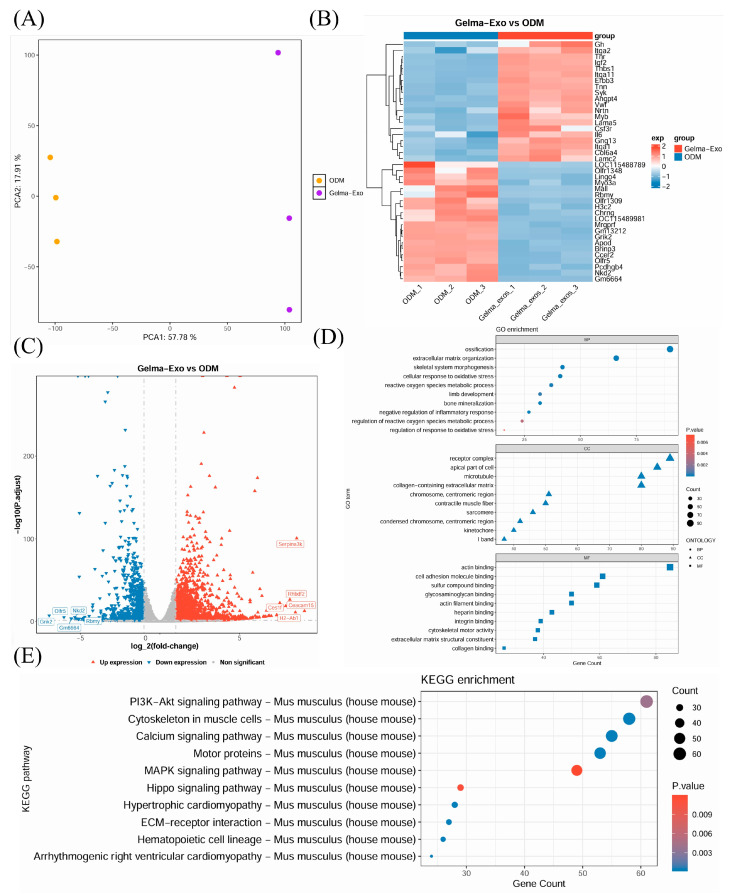
Transcriptomic profiling of MC3T3-E1 cells: GelMA@Exo vs. ODM. (**A**) Principal component analysis (PCA) plot showing distinct clustering of GelMA@Exo-treated cells vs. ODM control cells. (**B**) Heatmap of hierarchically clustered differentially expressed genes (DEGs) between GelMA@Exo and ODM, illustrating global expression changes. (**C**) Volcano plot of DEGs: red dots indicate upregulated genes and blue dots indicate downregulated genes (threshold: |log_2 fold-change| > 1, FDR < 0.05). (**D**) Gene Ontology (GO) enrichment analysis of upregulated genes (top categories in Biological Process, Cellular Component, Molecular Function). (**E**) KEGG pathway enrichment analysis showing top significantly enriched pathways upon GelMA@Exo treatment (e.g., PI3K-Akt signaling, MAPK, Hippo, etc., *p* < 0.05).

Global transcriptomic analysis provided insight into the broad genetic programs influenced by GelMA@Exo. Principal component analysis of RNA-seq data (Figure 9A) showed a clear separation between GelMA@Exo-treated samples and control samples, indicating distinct gene expression profiles.

In line with KEGG enrichment results, the PI3K-Akt pathway was significantly enriched in the GelMA@Exo group, implicating it as a key mediator in the observed bone-promoting effects. The proposed mechanistic diagram (Figure 9B) illustrates how multiple upregulated genes potentially activate PI3K/Akt signaling via integrin, IGF1R, and EGFR, ultimately enhancing the expression of osteogenic markers such as RUNX2 and ALP to promote differentiation and matrix mineralization.

Volcano plot analysis (Figure 9C) identified a large number of differentially expressed genes (DEGs) due to GelMA@Exo treatment: approximately 2566 genes met the criteria (|log_2 fold-change| > 1, FDR < 0.05), with roughly 1590 genes upregulated and 976 downregulated compared to ODM. Many of the upregulated genes are known to be involved in osteogenesis, extracellular matrix production, and cellular growth. For instance, GelMA@Exo treatment increased expression of key bone-regeneration genes such as spp.1 (osteopontin), Serpine1, Igfbp5, and Col1a1—genes that play roles in bone formation, matrix organization, and signaling—relative to ODM treatment.

Gene Ontology enrichment analysis of upregulated genes (Figure 9D) revealed significant over-representation of categories related to bone development, extracellular matrix organization, response to oxidative stress, and structural molecule activity, among others. This suggests GelMA@Exo creates a cellular environment geared toward building bone tissue and coping with stress (consistent with reduced senescence). Concurrently, KEGG pathway analysis (Figure 9E) indicated that GelMA@Exo activated numerous signaling pathways relevant to osteogenesis and tissue regeneration. In total, dozens of pathways were significantly enriched (*p* < 0.05), with some of the top upregulated pathways including PI3K-Akt signaling, calcium signaling, MAPK signaling, Hippo signaling, and ECM–receptor interaction pathways. These pathways are integral to cell proliferation, survival, differentiation, and matrix formation, reinforcing the view that GelMA@Exo engages multiple biological processes to promote osteogenic differentiation.

In summary, the transcriptomic data confirm that GelMA@Exo induces extensive changes in gene expression that favor osteogenesis. By upregulating a wide array of osteogenic genes and activating key pathways (like PI3K/Akt and others), GelMA@Exo appears to orchestrate a multi-faceted pro-osteogenic program at the molecular level, which aligns with the observed phenotypic outcomes (enhanced differentiation, mineralization, etc.).

### 4.9. Activation of PI3K/Akt Signaling Mediates GelMA@Exo’s Osteogenic Effect

**Figure 10:**
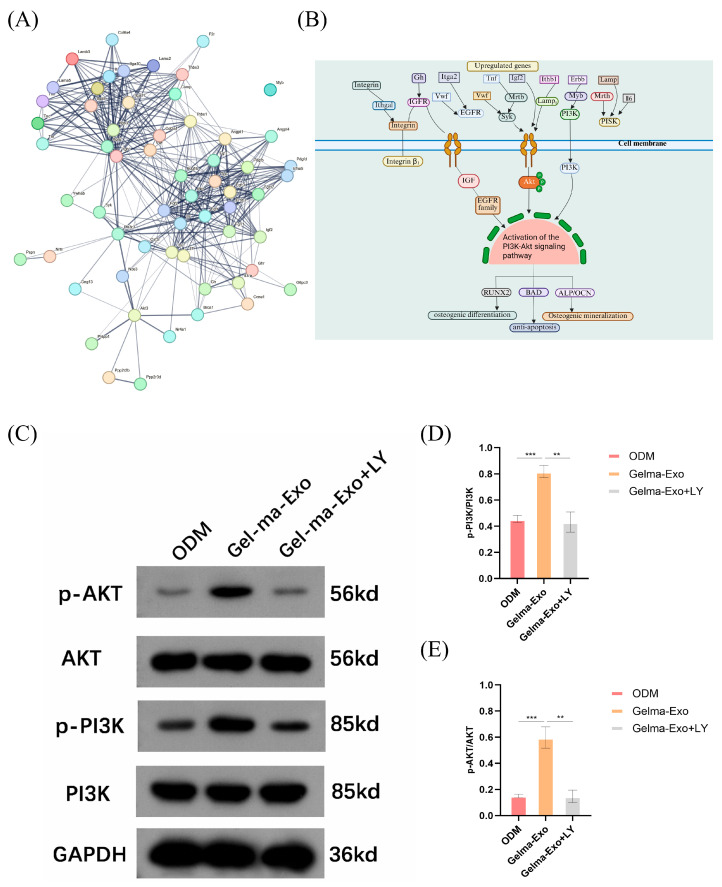
PI3K/Akt pathway activation by GelMA@Exo and its role in osteogenesis. (**A**) Protein–protein interaction (PPI) network analysis of DEGs (based on STRING) highlighting hub proteins (integrins, Akt1, TGFBR1, etc.) involved in osteogenic signaling. (**B**) Schematic summary of key upregulated signaling pathways (especially PI3K/Akt) and gene networks in GelMA@Exo-treated cells. (**C**) Western blot showing levels of phosphorylated PI3K (p-PI3K), total PI3K, phosphorylated Akt (p-Akt), and total Akt in MC3T3-E1 cells under ODM, GelMA@Exo, and GelMA@Exo + PI3K inhibitor (LY294002) conditions (GAPDH as loading control). (**D**,**E**) Densitometric analysis of p-PI3K/PI3K (**D**) and p-Akt/Akt (**E**) ratios (mean ± SD, n = 3). GelMA@Exo significantly increases PI3K/Akt activation compared to ODM (*p* < 0.001); LY294002 significantly reduces this activation (*p* < 0.01 vs. GelMA@Exo), indicating that GelMA@Exo’s pro-osteogenic effect may be mediated through PI3K/Akt signaling. Statistical analysis: one-way ANOVA with Tukey’s post hoc test. Significance: *** *p* < 0.001, ** *p* < 0.01.

To pinpoint crucial signaling mediators, we further analyzed protein interaction networks and performed targeted pathway validation. A STRING protein–protein interaction (PPI) network constructed from the DEGs revealed several hub nodes central to the osteogenic response (Figure 10A). Among these, integrin receptors (Itgb1 for β1 and Itga5 for α5) stood out; integrin α5β1 is known to activate FAK/PI3K/Akt pathways, which drive osteoblast differentiation and survival. Other key nodes included Akt1, Tgfbr1 (TGF-β type I receptor), Egfr (epidermal growth factor receptor), Fgfr1 (fibroblast growth factor receptor 1), and extracellular matrix proteins like Col1a1, Fn1 (fibronectin), and laminin subunits [26]. The prominence of Akt1 in the network underscores the importance of PI3K/Akt signaling, as Akt1 is a pivotal kinase that influences RUNX2 activity and bone formation [27]. The presence of Tgfbr1 aligns with the observed TGF-β1 upregulation; TGF-β signaling through TGFBR1 and Smad proteins can promote Osterix and COL1A1 expression [28]. The inclusion of ECM components (Col1a1, Fn1, laminins) in the network suggests that GelMA@Exo not only triggers intracellular pathways, but also affects the extracellular environment and mechanotransduction (likely via integrin-mediated feedback loops) [29].

Figure 10B presents a schematic overview of the signaling pathways and gene networks upregulated in MC3T3-E1 cells treated with GelMA@Exo. The diagram emphasizes the PI3K/Akt signaling pathway, which is activated upon exposure to GelMA@Exo. Key upregulated genes such as integrins, TGF-β, IGF1R, and EGFR play crucial roles in this process. These molecules interact with the PI3K pathway, leading to phosphorylation of Akt, which subsequently enhances osteogenic differentiation. The activation of PI3K/Akt results in the upregulation of osteogenic markers (e.g., RUNX2, ALP), which are essential for osteogenesis and matrix mineralization. This pathway is pivotal in mediating the regenerative effects of GelMA@Exo, promoting a pro-osteogenic environment.

Based on the network and enrichment results, we focused on the PI3K/Akt pathway as a major mediator of GelMA@Exo’s biological effects. Western blot analysis demonstrated that GelMA@Exo treatment markedly increased the levels of phosphorylated PI3K (p85 subunit) and phosphorylated Akt in MC3T3-E1 cells compared to Cells treated with ODM (Figure 10C), suggesting activation of the PI3K/Akt pathway. To determine whether this activation was essential for the observed effects, we introduced the PI3K inhibitor LY294002. Notably, LY294002 significantly inhibited Akt phosphorylation, confirming effective suppression of the PI3K/Akt signaling pathway. In GelMA@Exo-treated cells exposed to LY294002, the phosphorylation of both PI3K and Akt was substantially reduced relative to untreated GelMA@Exo cells, indicating that the effects of GelMA@Exo may be dependent on this pathway. Quantification of band intensities (Figure 10D,E) further showed that GelMA@Exo significantly elevated the p-PI3K/PI3K and p-Akt/Akt ratios (*** *p* < 0.001 vs. ODM), whereas LY294002 brought these values down to near-control(ODM) levels (** *p* < 0.01 vs. GelMA@Exo without inhibitor). Taken together, these data support the possibility that “GelMA@Exo promotes MC3T3-E1 cell osteogenic differentiation through the PI3K/Akt Pathway.”

## 5. Conclusions

In this study, we systematically evaluated the biological effects and underlying mechanisms of a GelMA hydrogel loaded with Epimedium-derived exosomes (GelMA@Exo) on MC3T3-E1 preosteoblasts and HUVECs. Our findings demonstrated that the GelMA@Exo composite hydrogel greatly improved exosome stability and local delivery, and exerted synergistic effects to promote cell proliferation, migration, angiogenesis, anti-senescence, and osteogenic differentiation. Key conclusions are as follows:

(1) Epimedium-derived exosomes were roughly 90–130 nm in size with intact RNA and protein cargo, indicating high purity and stability. GelMA hydrogels (5–12.5% *w*/*v*) formed porous 3D networks with tunable pore size, swelling ratio, and degradation rate, making them suitable and controllable scaffolds for delivering bioactive factors.
(2) GelMA hydrogels enabled sustained exosome release for up to 7 days in a concentration-dependent manner. The released exosomes were uniformly distributed throughout the hydrogel and efficiently internalized by MC3T3-E1 cells, demonstrating that the GelMA@Exo system has high delivery efficiency and excellent biocompatibility.
(3) GelMA@Exo synergistically enhanced multiple cellular functions. MC3T3-E1 proliferation increased with exosome dose (peaking at 195 μg/mL), and the combination of 5% GelMA + 195 μg/mL exosomes produced a strong synergistic proliferative effect (whereas GelMA alone had minimal impact). Notably, the GelMA@Exo composite Compared with other groups significantly improved angiogenesis (tube formation by HUVECs) and cell migration (scratch wound closure), highlighting its promise for enhancing tissue regeneration.
(4) GelMA@Exo promoted osteogenic differentiation. It significantly elevated early ALP activity and later matrix mineralization (Alizarin Red staining) in MC3T3-E1 cells. Molecular analyses corroborated these results: GelMA alone did not upregulate osteogenic markers, exosomes alone did to some extent, and GelMA@Exo further amplified the expression of key osteogenic genes/proteins (RUNX2, Osterix, COL1A1, ALPL, TGF-β1). contributing to the composite’s enhanced osteogenic effect and fostering a more regenerative microenvironment.
(5) Anti-senescence: GelMA@Exo exhibited a potent anti-senescent effect—treated osteoblasts had markedly fewer SA-β-Gal-positive senescent cells compared to ODM. Mechanistically, transcriptomic profiling showed broad upregulation of osteogenesis-related genes in GelMA@Exo-treated cells. Western blot analysis revealed elevated phosphorylation of PI3K and Akt following GelMA@Exo treatment, indicating activation of the PI3K/Akt signaling pathway. Moreover, treatment with the specific PI3K inhibitor LY294002 significantly suppressed Akt phosphorylation, confirming the involvement of the PI3K/Akt pathway in this process. Collectively, these results suggest that GelMA@Exo may facilitate the formation of an osteogenic microenvironment via activation of the PI3K/Akt signaling cascade.
(6) Overall: We developed a GelMA@Exo composite hydrogel that synergistically provides structural support and osteoinductive stimulation, creating a robust pro-osteogenic microenvironment. This system enhanced the osteogenic differentiation of MC3T3-E1 cells by acting as a supportive 3D scaffold while concurrently delivering bioactive exosomes that modulate gene expression. Plant-derived exosome-loaded hydrogels could be a viable translational strategy for bone tissue engineering and regenerative medicine, possibly through activation of the PI3K/Akt signaling pathway.

## 6. Future Perspectives

GelMA hydrogels (printable, mechanically tunable, and cytocompatible) [30,31,32], together with plant-derived exosomes (low immunogenicity, high stability, efficient uptake) [33], represent a promising cell-free therapeutic platform in regenerative tissue engineering.

In vitro, GelMA@Exo promoted osteogenesis, reduced cellular senescence, and enhanced angiogenesis. The next step is to validate these benefits in vivo. It remains uncertain whether the GelMA@Exo system will maintain its integrity, sustained exosome release, and bioactivity in the complex in vivo environment. Therefore, preclinical animal studies are needed to confirm that GelMA@Exo can integrate into host tissue, continuously deliver exosomes, and induce bone regeneration under physiological conditions.

Critical-size bone defect models in animals (rats, rabbits, etc.) are needed to test GelMA@Exo’s bone-healing efficacy, exosome release profile, and tissue integration in vivo [34,35]. New bone formation and neovascularization should be assessed by micro-CT, histology, and immunohistochemistry [36]. Additionally, the scaffold’s biodegradation, biocompatibility, and potential immune responses must be monitored to ensure safety and clinical feasibility.

The GelMA@Exo scaffold could be further optimized by incorporating functional peptides (e.g., RGD or bone-targeting motifs) to improve bone targeting and cell attachment/osteogenesis [37]. Moreover, advanced biofabrication techniques (e.g., microfluidics or 3D bioprinting) could be employed to precisely control scaffold architecture and exosome distribution, enabling patient-specific bone repair solutions [38]. Furthermore, because exosome cargo varies with the plant source, thorough characterization of Epimedium exosomes (e.g., small RNA sequencing, proteomics) is required to understand how their contents contribute to therapeutic effects [39,40]. In addition, integrating “smart” stimuli-responsive elements into the hydrogel (sensitive to pH, enzymes, temperature, etc.) could enable more controlled, on-demand exosome release for improved timing and targeting of therapy [41].

Translation to clinic: To move this approach toward clinical use, scalable, GMP-compliant production of plant-derived exosomes must be established, along with rigorous characterization to ensure consistent potency, purity, and safety [42]. Clear regulatory guidelines will also be needed to support future clinical trials of exosome-based therapies.

Overall, GelMA-based plant exosome delivery platforms offer a tunable, multi-functional strategy with considerable translational potential. With continued optimization—including in vivo validation, advanced manufacturing/biofabrication, and defined clinical development pathways—the GelMA@Exo system could become a safe and effective regenerative solution for challenging bone defects.

## Data Availability

The datasets generated and/or analyzed during the current study are available from the corresponding authors upon reasonable request.
